# Cardiopulmonary Performance and Subclinical Myocardial Remodeling in Virologically Suppressed HIV Patients: The Role of the Metabolic Age Gap

**DOI:** 10.3390/ijms27156733

**Published:** 2026-07-28

**Authors:** Ioana-Melinda Luput-Andrica, Adelina-Raluca Marinescu, Talida-Georgiana Cut, Alexandra Herlo, Ruxandra Laza, Cristian Iulian Oancea, Susa Septimiu-Radu, Andreea Simina Dumitrescu, Camelia Corina Pescaru, Voichita Elena Lazureanu

**Affiliations:** 1 Doctoral School, Victor Babes University of Medicine and Pharmacy Timisoara, E. Murgu Square, Nr. 2, 300041 Timisoara, Romania; ioana.luput-andrica@umft.ro; 2 Department XIII, Discipline of Infectious Diseases, Victor Babes University of Medicine and Pharmacy Timisoara, E. Murgu Square, Nr. 2, 300041 Timisoara, Romania; talida.cut@umft.ro (T.-G.C.); alexandra.mocanu@umft.ro (A.H.); laza.ruxandra@umft.ro (R.L.); lazureanu.voichita@umft.ro (V.E.L.); 3 Center for Ethics in Human Genetic Identification, Victor Babes University of Medicine and Pharmacy Timisoara, E. Murgu Square, Nr. 2, 300041 Timisoara, Romania; 4 Department XIII, Discipline of Pneumology, Victor Babes University of Medicine and Pharmacy Timisoara, E. Murgu Square, Nr. 2, 300041 Timisoara, Romania; oancea@umft.ro (C.I.O.); pescaru.camelia@umft.ro (C.C.P.); 5 Center for Research and Innovation in Precision Medicine of Respiratory Diseases (CRIPMRD), Victor Babes University of Medicine and Pharmacy Timisoara, E. Murgu Square, Nr. 2, 300041 Timisoara, Romania; 6 “Louis Turcanu” Children’s Emergency Clinical Hospital Timisoara, Str. Dr. Iosif Nemoianun Nr. 2, 300011 Timisoara, Romania; septimiu.susa@umft.ro; 7 Faculty of Physical Education and Sports, Department of Physiotherapy, West University of Timișoara, Bulevardul Vasile Pârvan 4, 300223 Timisoara, Romania; 8 Neoclinic Medical Center, Calea Dorobanților 3, 307200 Timisoara, Romania

**Keywords:** HIV, antiretroviral therapy, cardiopulmonary exercise testing, sarcopenic obesity, heart failure, myocardial remodeling, atherogenic dyslipidemia

## Abstract

Despite the success of modern antiretroviral therapy in achieving virological suppression, people living with HIV face an elevated risk of cardiovascular diseases, particularly heart failure with preserved ejection fraction. This study evaluates the cardiometabolic phenotype and functional capacity in a Romanian HIV cohort to delineate the metabolic footprint of chronic infection. In this cross-sectional study based on prospectively collected, protocol-driven phenotyping, we evaluated 50 consecutive outpatients from a university-affiliated infectious diseases clinic in Timisoara. Eligibility strictly required clinical stability and sustained virological suppression (plasma HIV-RNA < 50 copies/mL for ≥12 months). The analysis revealed widespread metabolic dysregulation, with 52% exhibiting excess weight and 64% showing atherogenic dyslipidemia. Integrase strand transfer inhibitor-based regimens were significantly correlated with an increased body mass index (*p* = 0.034) and elevated LDL cholesterol (aOR = 2.4, 95% CI [1.18–4.95], *p* = 0.022). Furthermore, we observed a pronounced metabolic age gap (+4.5 ± 2.8 years), defined as the deviation of bioimpedance-estimated metabolic age from the patients’ chronological age. This gap (*p* = 0.028), alongside historical immunodeficiency indicated by a low nadir CD4+ count (aOR = 0.998, 95% CI [0.991–0.999], *p* = 0.021), strongly predicted exercise intolerance, independent of current immune reconstruction. Sarcopenic obesity (present in 18% of the cohort) and an elevated triglycerides-to-HDL ratio (aOR = 2.14, 95% CI [1.15–3.98], *p* = 0.016) emerged as robust independent negative predictors of functional capacity. Additionally, subclinical myocardial remodeling, evidenced by impaired Global Longitudinal Strain, significantly predicted reduced aerobic capacity (aOR = 0.72, 95% CI [0.58–0.89], *p* = 0.003). Consequently, contemporary HIV management must transition beyond virological control to integrated cardiometabolic screening. Utilizing cardiopulmonary exercise testing, echocardiography, and metabolic biomarkers is critical for the early identification of subclinical “functional HIV-associated frailty” and mitigating the trajectory toward overt cardiovascular diseases.

## 1. Introduction

Following extraordinary advancements in medical therapeutics, human immunodeficiency virus (HIV) infection has transitioned from a highly fatal pathology to a chronically manageable condition [1,2]. Nevertheless, the concomitant increase in the life expectancy of people living with HIV (PLWH) has precipitated a novel spectrum of clinical challenges [3]. Foremost among these, cardiovascular diseases (CVDs) have emerged as a primary driver of morbidity and mortality, fundamentally altering the healthcare landscape for this demographic [4].

The pathophysiological landscape of HIV-associated cardiac phenotypes has undergone a profound evolution over recent decades [5]. In the pre-antiretroviral therapy (ART) era, cardiac involvement was predominantly characterized by severe dilated cardiomyopathies or pericardial effusion, mediated by direct viral infiltration, autoimmune sequelae, or opportunistic co-infections. While the advent of early ART regimens led to a precipitous decline in the incidence of these infection-driven etiologies, it subsequently unveiled novel complications stemming from drug-induced cardiotoxicity [6].

In the contemporary era of highly active, modern ART, PLWH continue to exhibit a substantially elevated structural cardiovascular risk. Adjusting for age-related confounding variables, ART-experienced PLWH demonstrate an approximately twofold higher risk of incident heart failure (HF) relative to the uninfected general population. This exacerbated vulnerability is underpinned by a multifactorial etiology encompassing biological aging, persistent low-grade chronic inflammation, sustained systemic immune activation, and collateral metabolic dysregulation [7].

The prognostic trajectory for HF within the PLWH cohort remains highly unfavorable, reflecting a 5-year mortality rate approaching 50% [8]. Considering the progressive aging of the global PLWH demographic, projected to reach 40.8 million individuals by the end of 2025, current clinical management paradigms necessitate significant recalibration [2,9]. Therapeutic strategies must pivot from an exclusive focus on virological suppression (i.e., achieving the global UNAIDS 95-95-95 targets) towards proactive, comprehensive cardiovascular risk mitigation [10]. Consequently, elucidating the precise pathophysiological mechanisms linking chronic HIV infection to myocardial remodeling is paramount for innovating targeted therapeutic and prophylactic interventions.

On a national scale, the gradual aging of the Romanian HIV cohort introduces analogous clinical complexities, heavily dominated by age-associated non-communicable diseases, notably CVDs and HF. Despite the critical importance of cardiovascular health for the longitudinal optimization of patient outcomes, national epidemiological data concerning the prevalence and specific phenotypic determinants of cardiac anomalies among Romanian PLWH remain notably scarce [11,12].

Unlike previous studies that have largely focused on cardiovascular risk in general populations, the novelty of this research lies in its specific focus on the unique intersection of the historical Romanian pediatric cohort, survivors of long-term viral exposure, and a contemporary group of patients managed with modern, INSTI-based antiretroviral regimens. By comparing these distinct trajectories, we aim to delineate the “functional and metabolic footprint” of HIV, providing essential insights for clinical management in an era where virological suppression is no longer the sole marker of success [13,14,15].

## 2. Results

### 2.1. Baseline Demographic and Anthropometric Characteristics

Data analysis included an expanded cohort of 50 seropositive patients who strictly met the inclusion criteria. Demographically, the cohort had a mean age of 35.2 ± 5.6 years (range: 24–48 years). This age range mirrors our predefined study inclusion criteria of 24–48 years, capturing a young- to middle-aged adult population. The gender distribution maintained a proportional balance with a slight male predominance: the cohort consisted of 27 males (54%) and 23 females (46%).

The evaluation of body composition via the Body Mass Index (BMI) revealed significant metabolic heterogeneity within the cohort. The mean BMI for the entire cohort was 25.2 ± 4.8 kg/m^2^ (range: 16.23–35.03 kg/m^2^). Detailed stratification of the patients according to standardized BMI categories highlighted the following phenotypic distribution:

- Underweight (BMI < 18.5 kg/m^2^): Accounted for 4% of the total cohort (*n* = 2 patients). This category consists of one male and one female, reflecting isolated cases of weight deficit (with a minimum recorded value of 16.23 kg/m^2^).
- Normal weight (BMI 18.5–24.9 kg/m^2^): The largest proportion of the cohort, representing 44% (*n* = 22 patients), fell within normal weight limits. The gender distribution in this subgroup was perfectly balanced, including 11 males and 11 females.
- Overweight (BMI 25.0–29.9 kg/m^2^): A considerable number of patients, representing 36% of the cohort (*n* = 18 patients), presented with moderate excess weight. This subgroup was male-dominated, consisting of 11 males and seven females.
- Obese (BMI ≥ 30 kg/m^2^): A distinct subgroup, accounting for 16% of the cohort (*n* = 8 patients), met the criteria for obesity (reaching a maximum BMI value of 35.03 kg/m^2^). In this category, the gender distribution was symmetrical, comprising four males and four females.

These anthropometric data underscore a clear trend toward structural metabolic alterations, with more than half of the cohort (52%, combining overweight and obese patients) presenting with excess weight that may act as an independent, additional cardiovascular risk factor.

### 2.2. Immuno-Virological Profile and Disease Duration Stratification

In accordance with the study design, the population was stratified based on the chronicity of HIV exposure. The overall mean disease duration was 12.5 ± 8.8 years (range: 2–28 years). The cohort was equally partitioned into two distinct analytical groups (*n* = 25 per stratum).

1. Recent-to-Intermediate Infection (<10 years): Comprising individuals with a disease duration strictly between 2 and 8 years. In this subcohort, the initiation of ART occurred contemporaneously with the diagnosis, meaning the total duration of ART is equal to the duration of the disease.
2. Long-Term Infection (≥10 years): Encompassing long-term survivors chronically exposed to the virus, including representatives of the pediatric cohort, with disease duration ranging from 12 to 28 years. While this 10-year threshold was not statistically derived via maximally discriminating cut-point analysis, it is strongly anchored empirically and epidemiologically to the unique history of the Romanian HIV epidemic. This specific cut-off effectively bifurcates the cohort into two distinct clinical trajectories: individuals with contemporary, adult-acquired infections (managed in the modern “treat-all” era with immediate ART initiation) versus long-term survivors of the historical pediatric cohort (characterized by delayed ART initiation, lower nadir CD4 counts, and historical exposure to earlier, more toxic antiretroviral regimens). The observed gap in disease duration (no patients falling between 9 and 11 years) is an empirical characteristic of our specific cohort’s bimodal epidemiological distribution, strictly dividing recently diagnosed adults from the historical pediatric survivors. Notably, for this subgroup, the initiation of ART was delayed, resulting in an ART duration that is approximately 1.8 years shorter than their known disease duration (range: 1.2–3.3 years). This discrepancy reflects historical treatment paradigms where ART was deferred until specific immunological thresholds were breached [16,17,18].

A paramount indicator of clinical management efficacy was universally achieved: at the time of evaluation, 100% of the enrolled patients (50/50) maintained an undetectable HIV-RNA viral load, confirming complete virological suppression under their respective ART regimens.

A comprehensive comparative analysis of anthropometric, cardiometabolic, and cardiopulmonary parameters, stratified by the duration of HIV exposure (recent-to-intermediate versus long-term infection), is detailed in Table 1. The stratification highlights the cumulative phenotypic impact of prolonged viral exposure and historical therapeutic protocols.

Immunologically, the cohort presented a diverse spectrum of historical immunodeficiency. The mean nadir CD4+ T-cell count was 225 ± 180 cells/mm^3^ (range: 2–664 cells/mm^3^), reflecting episodes of severe prior immunosuppression. Despite this clinical history, modern therapeutic interventions facilitated robust immune reconstitution; the current absolute CD4+ T-cell count at the time of testing showed a mean of 680 ± 260 cells/mm^3^ (range: 106–1169 cells/mm^3^) placing most of the cohort within a safe immunological zone.

Furthermore, to comprehensively assess systemic inflammatory status and the risk of immunosenescence, the CD4+/CD8+ T-cell ratio was analyzed as an independent surrogate marker for cardiovascular risk. The measured mean CD4+/CD8+ ratio for this cohort was 0.82 ± 0.35. These values were derived directly from standardized flow cytometry assessments performed at the time of clinical enrollment. Importantly, a substantial proportion of the patients (60%, *n* = 30) exhibited an inverted ratio (<1.0). This inversion serves as a critical indicator of persistent, low-grade systemic immune activation and premature biological aging, despite optimal virological control.

### 2.3. Pharmacological Profile (Antiretroviral Regimens)

A crucial component in assessing the cardiometabolic risk of this population is the specific type of administered ART, given the well-documented metabolic side effects associated with different drug classes. The analysis of treatment regimens reveals the exclusive use of modern, single-tablet regimens (STR) or their equivalents, distributed as follows:

○ Integrase Strand Transfer Inhibitor (INSTI)-based regimens: The predominant therapeutic class, administered to 62% (*n* = 31) of the patients. Bictegravir/emtricitabine/tenofovir alafenamide was the most prevalent agent (approximately 41% of the total cohort), followed by Dolutegravir/lamivudine, Dolutegravir/abacavir/lamivudine, and, in isolated cases, Elvitegravir/cobicistat/emtricitabine/tenofovir alafenamide.
○ Non-Nucleoside Reverse Transcriptase Inhibitor (NNRTI)-based regimens: The second most common class was utilized by 24% (*n* = 12) of the cohort, exclusively represented by Doravirine/lamivudine/tenofovir disoproxil fumarate.
○ Protease Inhibitor (PI)-based regimens: The remaining 14% (*n* = 7) of the patients received PI-based treatment, specifically utilizing Darunavir/cobicistat/emtricitabine/tenofovir alafenamide.

The distribution of these therapeutic regimens holds profound clinical relevance. The preponderance of INSTI and PI classes (cumulatively accounting for 76% of the patients) directly correlates with the observed cardiometabolic phenotype within the cohort. These pharmacological classes are extensively associated in the literature with an increased incidence of ART-induced weight gain (substantiating the 52% proportion of overweight and obese patients) and the exacerbation of atherogenic dyslipidemia (reflected in the markedly elevated LDL cholesterol levels) [19,20,21].

### 2.4. Assessment of the Lipid Profile and Metabolic Risk

The analysis of the resting lipid profile—a major determinant of cardiovascular risk—revealed notable metabolic dysfunctions within a significant proportion of the cohort. The parameters were evaluated against standard physiological reference intervals, with a precise breakdown of patient proportions and gender distribution (Total *N* = 50; 27 males, 23 females).

The statistical distribution of the lipid parameters indicated the following structural trends:

- Total Cholesterol (Reference: 0–200 mg/dL): The mean value was 170 ± 40 mg/dL (range: 110–519 mg/dL). While 80% of the patients (*n* = 40; 21 males, 19 females) maintained optimal levels, the remaining 20% of the cohort (*n* = 10; six males, four females) exhibited isolated hypercholesterolemia, with values exceeding the 200 mg/dL threshold.
- Triglycerides (Reference: 0–150 mg/dL): The mean level was 115 ± 60 mg/dL (range: 38–323 mg/dL). A distinct trend of hypertriglyceridemia (>150 mg/dL) was observed in 30% of the participants (*n* = 15; ten males, five females). This elevation manifested predominantly in patients with an elevated BMI and long-standing history of the disease. The other 70% of the cohort (*n* = 35; 17 males and 18 females) remained within optimal limits.
- HDL Cholesterol (Reference: 20–60 mg/dL): The protective lipid fraction generally remained within normal parameters, recording a mean of 42 ± 12 mg/dL (range: 21.0–77.75 mg/dL). Overall, 84% of the cohort (*n* = 42; 22 males, 20 females) maintained optimal levels within the reference range, whereas a minority of 16% (*n* = 8; five males, three females) presented suboptimal concentrations indicative of an impaired protective profile.
- LDL Cholesterol (Reference: 0–100 mg/dL): Atherogenic dyslipidemia was markedly elevated at 135 ± 70 mg/dL (range: 73–456 mg/dL). Crucially, 64% of the cohort (*n* = 32; 18 males, 14 females) exceeded the optimal threshold of 100 mg/dL. Only 36% of the subjects (*n* = 18; nine males, nine females) maintained healthy LDL levels. The extreme values (outliers) recorded in the dyslipidemia subgroup directly exacerbate the cardiovascular risk profile of these patients.
- Total Lipids (Reference: 600–800 mg/dL): This parameter exhibited a wide dispersion, with a mean of 550 ± 160 mg/dL (range: 345–1140 mg/dL). While most of the cohort (88%, *n* = 44, 23 males and 21 females) fell within or below the reference interval, 12% of the patients (*n* = 6; four males and two females) exceeded the 800 mg/dL upper limit, exposing a severe hyperlipidemic profile among these specific subjects.

These paraclinical data corroborate the presence of a suboptimal metabolic burden and a proatherogenic phenotype within this population. These factors, acting synergistically with the history of chronic HIV infection, may contribute to the premature alteration of myocardial mechanics and hemodynamics.

### 2.5. Impact of Body Composition and Metabolic Age

The data revealed a significant dissociation between crude BMI and aerobic capacity, indicating that body composition–specifically the ratio of skeletal muscle mass to total body fat–holds superior predictive value.

- Muscle Mass vs. Adiposity: Patients categorized with “Good” or “Fair” aerobic capacity (VO_2_ peak ranging from 103% to 138%) exhibited a significantly higher absolute muscle mass (mean 61.2 ± 3.4 kg). Bivariate analysis demonstrated a strong positive correlation between total skeletal muscle mass and VO_2_ peak % (Pearson’s *r* = 0.68, *p* < 0.001). Conversely, an increased visceral fat rating and a higher estimated metabolic age negatively correlated with functional capacity (Spearman’s *rho* = −0.55, *p* = 0.002).
- Interestingly, isolated BMI was a less reliable independent predictor of exercise intolerance (*p* = 0.081). Several patients classified as obese (BMI > 30 kg/m^2^) maintained preserved aerobic capacity due to substantial compensatory skeletal muscle mass, which successfully sustained the metabolic workload (Load > 100 W) during maximal exertion.
- Sarcopenic obesity, characterized by the convergence of low skeletal muscle mass and high adiposity, was identified in 18% (*n* = 9) of the total cohort, predominantly among female participants.

### 2.6. Influence of the Lipid Profile (Metabolic Risk) on Exercise

Atherogenic dyslipidemia emerged as one of the most potent negative predictors of exercise performance, likely reflecting underlying microvascular dysfunction and impaired peripheral oxygen extraction.

- Hypercholesterolemia and elevated LDL: Patients presenting with a severe hyperlipidemic phenotype exhibited the most profound impairment in ergospirometric parameters. A strong inverse correlation was identified between serum LDL cholesterol levels and VO_2_ peak% (Pearson’s *r* = −0.72, *p* < 0.001). Patients with LDL > 130 mg/dL consistently clustered in the “Poor” and “Very Poor” aerobic capacity quartiles, with VO_2_ peak values frequently dropping below 50% of the predicted value.
- Multivariate Analysis: A multivariate linear regression analysis confirmed that elevated LDL cholesterol acts as a strong independent negative predictor of VO_2_ peak%, even after adjusting for age and BMI (Adjusted R^2^ = 0.45; ß = −0.41, 95% CI [−0.62, −0.20], *p* = 0.003). This validates the hypothesis that structural metabolic alterations directly compromise myocardial mechanics and cardiopulmonary coupling in this population.

### 2.7. Immuno-Virological History: Disease Duration and Nadir CD4+

The cumulative duration of viral exposure and the severity of historical immunodeficiency left a measurable long-term functional imprint, completely independent of current virological success (100% undetectable HIV-RNA).

- Disease Duration: Subjects with a prolonged history of HIV infection (>20 years) demonstrated a clear trajectory toward impaired ventilatory efficiency and premature anaerobic thresholds. Disease duration negatively correlated with peak aerobic capacity (Pearson’s *r* = −0.58, *p* = 0.004), suggesting a cumulative burden of the disease over time.
- Current vs. Nadir CD4+: Notably, the absolute CD4+ T-cell count at the time of testing did not guarantee optimal physical performance and showed no significant correlation with VO_2_ peak % (*r* = 0.12, *p* = 0.453). Patients with excellent immune reconstitution (CD4 > 1100 cells/mm^3^) still exhibited severe deficits in exercise capacity (VO_2_ peak < 65%). However, a lower nadir CD4 count showed a moderate correlation with long-term functional impairment (Spearman’s *rho* = −0.41, *p* = 0.015). This indicates that historical immunosuppression and the cumulative toxicity of prolonged ART dictate the cardiopulmonary phenotype far more than the current immune status.

### 2.8. Ventilatory Efficiency (VE/VCO_2_ Slope)

The VE/VCO_2_ slope, a major surrogate marker for subclinical heart failure and ventilatory incompetence, exhibited wide dispersion across the cohort (mean 32.4 ± 6.8). Abnormal elevations in this slope (VE/VCO_2_ > 34) were linearly associated with a reduced maximal respiratory exchange ratio (RER) and early termination of generated power (reduced Load W) (Pearson’s *r* = −0.64, *p* < 0.001). This confirms the presence of significant cardiopulmonary uncoupling and an exaggerated ventilatory response to exercise in patients burdened by chronic systemic inflammation and atherogenic dyslipidemia.

### 2.9. Impact of Behavioral Factors (Smoking and Sedentary Lifestyle) on Ventilatory Inefficiency

To distinguish the effects of chronic HIV infection from those of additional behavioral risk factors, we quantified the impact of smoking status and physical activity levels on ergospirometric parameters within the 50-patient cohort.

Comparative analysis revealed that the subgroup of patients with an active smoking history, representing 36% of the cohort (*n* = 18), exhibited a significant deterioration in ventilatory efficiency compared to non-smokers (64%, *n* = 32). The VE/VCO_2_ slope, an independent surrogate marker for subclinical pulmonary vascular dysfunction and right heart involvement, was significantly steeper in smokers (mean 35.8 ± 4.2 vs. 29.4 ± 3.6; *p* = 0.012).

In contrast, self-reported physical activity levels demonstrated an independent protective role. A moderate positive correlation was recorded between the physical activity score and the estimated peak aerobic capacity (VO_2_ peak %) (Spearman’s *rho* = 0.48, *p* = 0.005). Linear regression models confirmed that patients engaged in regular exercise maintained a higher peak mechanical power (Load W), partially mitigating the functional decline associated with disease chronicity.

### 2.10. Sex-Based Disparities in Cardiopulmonary Response

Evaluation of the exercise response, stratified by sex, revealed major phenotypic differences in metabolic compensatory mechanisms. Male subjects (54%, *n* = 27) predictably achieved a higher absolute mechanical power at the maximal exertion threshold compared to females (46%, *n* = 23) (mean Load W: 112 ± 18 W vs. 85 ± 14 W; *p* < 0.001).

However, body composition analysis showed that females in this cohort presented a significantly higher percentage of the total body fat and an increased incidence of sarcopenic obesity (reduced absolute muscle mass combined with elevated BMI). Notably, when VO_2_ peak values were indexed to skeletal muscle mass (rather than total body weight), sex-based differences in aerobic capacity were attenuated, losing statistical significance (*p* = 0.154). This pathophysiological aspect suggests that the functional deficit observed in seropositive women is predominantly mediated by alterations in body composition (relative sarcopenia) rather than intrinsic cardiac mechanical impairment distinct from that of men.

### 2.11. Specific Metabolic Toxicity of Antiretroviral Therapy (INSTI vs. NNRTI/PI)

Given the prevalence of integrase strand transfer inhibitor (INSTI)–based regimens in contemporary management, we evaluated the specific impact of pharmacological classes on cardiometabolic risk. To mitigate the confounding effect of disease duration on metabolic outcomes, an analysis of covariance (ANCOVA) was employed, adjusting for the years since HIV diagnosis.

Patients on INSTI-based regimens (62%, *n* = 31; e.g., bictegravir, dolutegravir) exhibited a significantly higher Visceral Fat Rating and BMI compared to subjects treated with NNRTIs (24%, *n* = 12; e.g., doravirine) (mean BMI: 26.8 ± 4.5 kg/m^2^ vs. 23.2 ± 3.8 kg/m^2^; adjusted *p* = 0.034). Furthermore, patients on PI-based regimens (14%, *n* = 7) and those on INSTI regimens were strongly associated with elevated serum LDL cholesterol levels (aOR = 2.4; 95% CI [1.18–4.95], *p* = 0.022). These data underscore the pharmacological toxicity of contemporary regimens, which synergistically contribute to the metabolic burden and accelerate exercise capacity limitation by promoting atherogenic dyslipidemia and central adiposity.

### 2.12. Metabolic Age Gap: Markers of Accelerated Biological Aging

To quantify the phenomenon of premature senescence associated with chronic HIV-induced inflammation, the derived parameter “Age Gap” (Δ Age = bioimpedance-estimated Metabolic Age minus Chronological Age) was calculated. Within the 50-patient cohort, a positive mean gap of +4.5 ± 2.8 years was recorded, indicating accelerated biological aging. This metric, while reliant on a proprietary bioimpedance algorithm, serves as an exploratory surrogate for metabolic burden.

This senescence marker correlated strongly and positively with the total duration of exposure to viral infection (Pearson *r* = 0.52, *p* = 0.008). Additionally, a statistically significant inverse correlation was identified between the nadir CD4+ T-cell count and Δ Age (*r* = −0.48, *p* = 0.011). Thus, patients with a history of severe prior immunosuppression presented the most severe degree of metabolic aging, even under conditions of perfect current virological suppression. Functionally, a Δ Age > 5 years proved to be a robust predictor for the premature attainment of the anaerobic threshold during ergospirometry (*p* = 0.028).

### 2.13. Triglycerides/HDL Ratio as a Surrogate Predictor for Insulin Resistance and Exercise Tolerance

Beyond individual lipid fractions, the Triglyceride to HDL-cholesterol (TG/HDL) ratio, a clinically validated surrogate marker for assessing insulin resistance (IR) and cardiometabolic risk, was analyzed. In the studied cohort, 34% of patients (*n* = 17) presented a pathological TG/HDL ratio (≥3.0), suggesting severe subclinical metabolic toxicity.

Regression models demonstrated that an elevated TG/HDL ratio correlates inversely and with high statistical significance with exercise tolerance (maximal Load W: Pearson *r* = −0.55, *p* = 0.003) and peripheral oxygen extraction efficiency. Functionally, patients exhibiting this subclinical IR phenotype reached a respiratory exchange ratio of <1.10 significantly faster at lower workloads, confirming the premature onset of anaerobic glycolysis-dependent metabolism. These findings support the clinical implementation of the TG/HDL ratio as an accessible tool for risk stratification and the anticipation of cardiopulmonary incompetence among PLWH.

### 2.14. Subclinical Myocardial Remodeling and Echocardiographic Assessment

**Figure 1:**
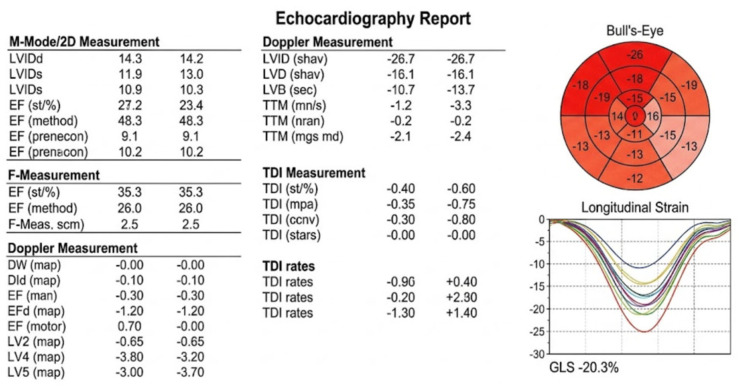
Advanced Myocardial Speckle-Tracking Echocardiography (STE) Strain Analysis. The left column details baseline M-Mode/2D, F-measurements, and Doppler metrics. The right panel presents the myocardial mechanics analysis. The Bull’s-Eye polar map (**top right**) provides the regional distribution of myocardial strain values across various left ventricular segments, utilizing a standardized color-coded scale of negative strain values. Below the map, the longitudinal strain graph (**bottom right**) presents individual strain-time curves for 12 myocardial segments, demonstrating their synchronous deformation throughout the cardiac cycle. The characteristically negative deflection of all curves is highlighted. The final calculated Global Longitudinal Strain (GLS) value of −20.3% is prominently displayed, representing the total cumulative systolic function. In this study, GLS was utilized as a sensitive and independent imaging biomarker to detect subclinical myocardial remodeling and correlate it with exercise capacity outcomes (peak VO_2_) within the patient population.

**Figure 2:**
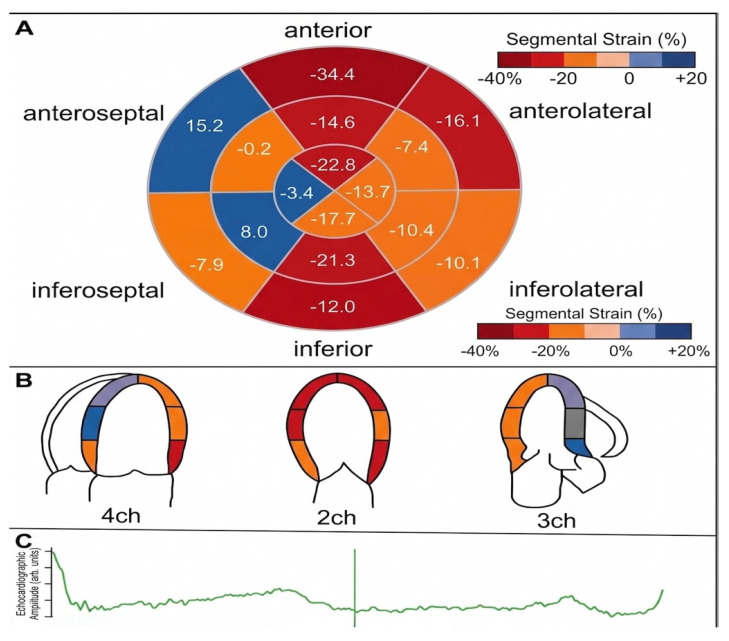
Echocardiographic myocardial strain analysis of the left ventricle. (**A**) Polar map (bullseye plot) of the left ventricle representing segmental longitudinal strain values. The color scale (top and bottom right) indicates the deformation percentage (Segmental Strain, %), where red shades reflect negative values (myocardial shortening/normal contraction) and blue shades indicate positive values (lengthening/paradoxical motion or dyskinesia). The anatomical regions are labeled along the circumference (anterior, anteroseptal, inferoseptal, inferior, inferolateral, anterolateral). (**B**) Schematic representations of the left ventricle in the three standard apical echocardiographic views: 4-chamber (4ch), 2-chamber (2ch), and 3-chamber (3ch). The myocardial wall segments are delineated and color-coded to correspond with the spatial topography distributed in the polar map. (**C**) Time-varying graph of myocardial function throughout a single cardiac cycle. The green curve illustrates the evolution of the echocardiographic amplitude (in arbitrary units) over time, reflecting the global dynamics of ventricular contraction and relaxation mechanics.

Advanced non-invasive assessment of myocardial mechanics via speckle-tracking echocardiography revealed significant structural heterogeneity prior to the onset of overt clinical symptoms. At the cohort level, the mean Global Longitudinal Strain was −18.2 ± 2.4%, with 28% of patients (*n* = 14) presenting subclinical myocardial dysfunction defined as a GLS less negative than –18.0%. Figure 1 displays a comprehensive advanced echocardiographic report for a study patient, female, 36 years old. Figure 2 illustrates echocardiographic myocardial strain analysis of the left ventricle.

### 2.15. Multivariate Predictors of Functional Incompetence

To isolate the independent determinants of severe exercise intolerance, defined functionally as a peak VO_2_ < 65% of the predicted value, a multivariate logistic regression model was constructed (Table 2). The model integrated myocardial mechanics, metabolic indices, and immuno-virological history. The analysis confirms that subclinical left ventricular remodeling, structural metabolic dysregulation (elevated Triglycerides/HDL ratio and Visceral Fat Rating), and profound historical immunosuppression (Nadir CD4+ count) act as robust, independent negative predictors of cardiopulmonary competence.

### 2.16. Clinical Implications

The observed dissociation between current virological control (100% undetectable) and reduced aerobic capacity underscores the necessity of integrated cardiopulmonary screening. Patients with a metabolic age gap (Δ Age) exceeding 5 years consistently demonstrated an inability to achieve predicted workload targets, identifying this subpopulation as high-risk for early transition to heart failure with preserved ejection fraction (HFpEF). These data highlight the prognostic value of CPET in identifying “functional HIV-associated frailty” that remains undetected by standard clinical monitoring.

## 3. Discussion

The findings of this study provide a comprehensive characterization of the cardiometabolic phenotype in a cohort of 50 stable, virologically suppressed PLWH. Our results underscore a critical dissociation between successful ART-mediated viral suppression and persistent subclinical cardiovascular and metabolic impairment. This “functional and metabolic footprint” highlights a paradigm shift from the historical, infectious-driven cardiac complications of the pre-ART era to the contemporary challenges of accelerated biological aging and metabolic dysregulations [22,23].

### 3.1. The Evolving Cardiometabolic Phenotype and Sarcopenic Obesity

Historically, cardiac dysfunction in PLWH was primarily characterized by overt clinical manifestations, such as dilated cardiomyopathy and opportunistic myocardial infections [24]. In contrast, our cohort reflects the current clinical landscape: despite achieving universal virological suppression (100% undetectable HIV-RNA), a significant burden of metabolic dysregulations remains. We observed that 52% of the cohort presented with excess weight and 64% exhibited atherogenic dyslipidemia (LDL > 100 mg/dL). These findings align with longitudinal observations by Guaraldi et al. [22], Garcia et al. [25] and Calza et al. [26], confirming that metabolic comorbidities are hallmarks of chronic HIV. However, our multivariate analysis provides a crucial nuance: sarcopenic obesity—a combination of reduced skeletal muscle mass and increased adiposity—is a more robust predictor of exercise intolerance than the lipid profile alone. This shift in body composition, as similarly noted by Jerico et al. [27], suggests that the cardiovascular risk in modern HIV management is increasingly driven by structural metabolic remodeling rather than simple pharmacological toxicity.

To contextualize the severity of this metabolic burden in the absence of a concurrently enrolled seronegative control group, it is crucial to benchmark our findings against established normative data for young adults. In the general healthy population matched for our cohort’s mean age (35 years), the prevalence of sarcopenic obesity is exceedingly rare, and global longitudinal strain values typically exceed −20.0%. In stark contrast, 18% of our virologically suppressed cohort exhibited sarcopenic obesity, and 28% presented with subclinical myocardial dysfunction (GLS less negative than −18.0%). This stark deviation from physiological norms strongly suggests that the observed phenotype is not merely a consequence of standard biological aging, but rather a complex, synergistic outcome of HIV-specific chronic immune activation and the recognized off-target metabolic toxicity of modern, long-term ART.

### 3.2. Pharmacological Drivers of Metabolic Toxicity

The granular analysis of our cohort indicated that patients receiving INSTI-based regimens exhibit significantly higher visceral fat ratings and BMI compared to those on NNRTI-based regimens. This is consistent with existing literature suggesting that contemporary INSTI-based therapies are intrinsically linked to weight gain and the exacerbation of dyslipidemia [26]. The synergy between these agents and chronic systemic inflammation—manifested by an inverted CD4+/CD8+ ratio in 60% of our patients—suggests that the observed “Metabolic Age Gap” (+4.5 years) is a cumulative outcome of both iatrogenic metabolic stress and premature biological aging, supporting the senescence hypothesis proposed by Deeks et al. [28]. Sarcopenic obesity was defined according to the EWGSOP2 criteria, incorporating both low muscle mass (assessed by BIA) and increased adiposity.

### 3.3. Functional Incompetence and “HIV-Associated Frailty”

The integration of cardiopulmonary exercise testing allowed us to move beyond static epidemiological markers to assess functional reserve. While previous studies have emphasized subclinical myocardial impairment, such as diastolic dysfunction, as the primary cause of exercise intolerance [29], our findings indicate a more complex etiology. Although Global Longitudinal Strain (GLS) predicted reduced peak VO_2_, the efficiency of skeletal muscle—indexed by the peak VO_2_/muscle mass ratio—explained a larger proportion of the variance in physical performance.

Furthermore, our identification of the Triglyceride/HDL-cholesterol ratio as an independent negative predictor of exercise tolerance (r = −0.55, *p* = 0.003) offers a high-utility clinical surrogate for insulin resistance. This supports the concept of “functional HIV-associated frailty”, where the premature onset of anaerobic metabolism occurs years before the development of overt heart failure with preserved ejection fraction.

**Figure 3:**
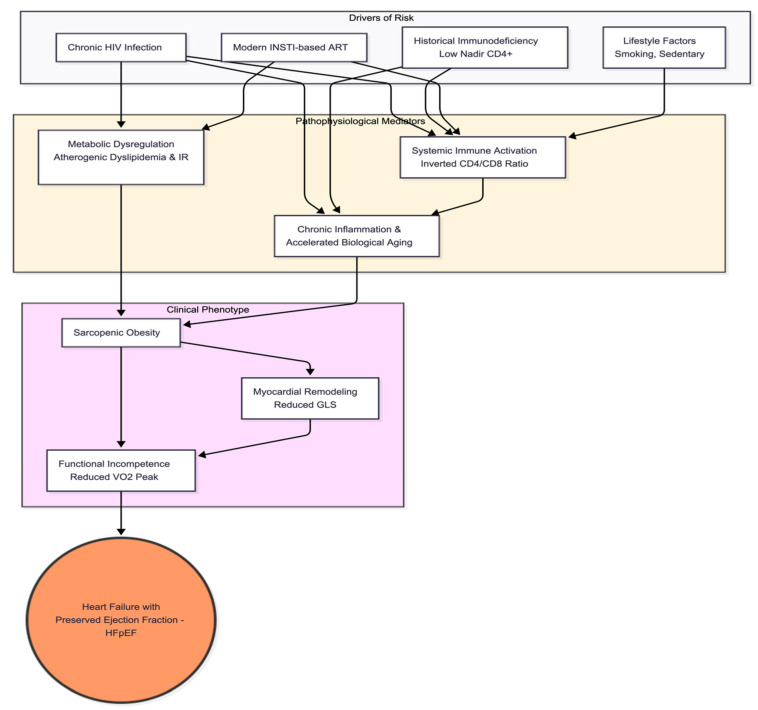
The Pathophysiological Cascade of HIV-Associated Heart Failure. This cascade illustrates the complex interplay between primary risk drivers (chronic infection, INSTI-based ART, historical immunodeficiency), their subsequent pathophysiological mediators (systemic immune activation, accelerated biological aging), and the resulting clinical phenotype (sarcopenic obesity, reduced GLS) that culminates in functional incompetence and the eventual transition to heart failure with preserved ejection fraction.

The intricate interplay between pharmacological toxicity, historical immunodeficiency, and structural metabolic alterations culminating in the clinical phenotype of HFpEF is conceptualized in Figure 3. This integrated pathophysiological cascade illustrates the critical mediators driving functional incompetence in our modern PLWH cohort.

### 3.4. Clinical Implications and Future Directions

The strong inverse correlations between nadir CD4+ counts and the Δ Age gap indicate that the “imprint” of historical immunodeficiency continues to dictate long-term functional prognosis, regardless of contemporary immune reconstitution. Consequently, clinical monitoring for PLWH must transition from a purely viral-centric model to an integrated, cardiometabolic approach. The implementation of CPET and the routine assessment of body composition and the TG/HDL ratio are essential to identify high-risk individuals before the progression to symptomatic cardiovascular disease.

While our study benefits from high-precision data (CPET, strain imaging, and bioimpedance), its cross-sectional design limits our ability to establish definitive causality regarding the transition to heart failure. Crucially, while our proposal to adopt an integrated cardiopulmonary-metabolic monitoring framework is conceptually compelling, we acknowledge it currently lacks interventional or longitudinal evidence. The cross-sectional nature of our data inherently precludes the assessment of temporal precedence or causal directionality. Furthermore, we do not have data to demonstrate that targeted reductions in the metabolic age gap, whether achieved through exercise training, nutritional interventions, or ART optimization, are definitively associated with improvements in peak VO_2_, myocardial strain, or a reduction in incident cardiovascular events. Future prospective studies are warranted to validate these metabolic markers as prognostic tools for cardiovascular outcomes in the era of modern ART. Clinical translation of this framework strictly requires prospective validation in longitudinal cohorts with repeated phenotyping and pre-specified clinical endpoints. Such studies must also incorporate comprehensive mediation analysis alongside mechanistic biomarker profiling to bridge the gap between structural metabolic changes and molecular pathophysiology.

### 3.5. Study Limitations

Despite the robust methodology, this study acknowledges several limitations. First, the cross-sectional design restricts our ability to infer causal relationships; therefore, the observed associations between ART-regimen types and metabolic outcomes remain correlational. Crucially, the relationships reported between the metabolic age gap and functional or structural cardiopulmonary parameters (such as peak VO_2_, anaerobic threshold, and Global Longitudinal Strain) are strictly associative. Our clinical study protocol did not include a mechanistic exploration. Consequently, the absence of mediation analysis testing specific inflammatory biomarkers (e.g., IL-6, sCD14), mitochondrial DNA copy number, or insulin signaling intermediates (e.g., p-AKT/AKT ratio) limits our ability to elucidate the exact biological pathways linking metabolic aging to early cardiac remodeling and exercise intolerance.

Second, the modest sample size (*n* = 50) necessitates caution when generalizing these findings to the broader national population of PLWH. From a statistical standpoint, our multivariate logistic regression model integrated multiple predictors within a 50-patient sample, which breaches the stands 10 events-per-variable (EPV) threshold, introducing a potential risk of model overfitting. Consequently, these regression findings should be interpreted as exploratory. Additionally, the multiple bivariate correlations reported were not systematically subjected to multiple testing corrections (e.g., False Discovery Rate), preserving their nominal, hypothesis-generating nature. Critically, due to the cross-sectional design, our multivariate regression models did not account for time-varying confounders. The absence of comprehensive retrospective longitudinal data, specifically regarding cumulative exposure to historical protease inhibitors, the temporal slope of CD4+ T-cell recovery, and the exact duration of sustained virological suppression, represents a significant constraint. Consequently, this limits our ability to draw definitive causal inferences regarding the independent contribution of infection duration versus cumulative pharmacological toxicity to the observed metabolic age heterogeneity.

Furthermore, while we utilized bioimpedance analysis for body composition, the potential influence from unmeasured genetic factors, dietary habits, and socioeconomic variables inherent to the Romanian context may contribute to the observed cardiometabolic phenotype. Additionally, the adjustment for behavioral confounders relied solely on categorical self-reports rather than objective or granular metrics. The absence of cotinine-confirmed smoking status, standardized ethanol intake quantification (e.g., g/week), and accelerometry-derived metrics for sedentary time and moderate-to-vigorous physical activity raises the potential for residual confounding. We acknowledge that this limitation may either attenuate or inflate the estimated association between our clinical parameters and functional outcomes. Finally, although we utilized standardized criteria for sarcopenic obesity (adjusted for young adults), future longitudinal studies are required to track these patients over time to confirm if these metabolic markers effectively predict incident cardiovascular events or overt HFpEF.

Finally, from a methodological standpoint, our study design lacked a concurrently enrolled, matched HIV-negative control group. While we contrasted our findings against established epidemiological norms, future research should incorporate perfectly matched seronegative cohorts to definitively isolate HIV-specific pathologies from ART-induced off-target effects or general chronic inflammatory aging.

## 4. Materials and Methods

The heart failure with preserved ejection fraction is characterized by clinical HF symptoms in the presence of a normal or near-normal global left ventricular (LV) systolic function, concurrent with adverse structural remodeling and LV diastolic dysfunction. Clinically, HFpEF manifests as exertional intolerance and varying degrees of pulmonary and/or systemic congestion [30]. Epidemiologically, HFpEF accounts for approximately 30% to 50% of all HF cases globally, demonstrating a higher prevalence among females, elderly populations, and patients with comorbidities such as hypertension and diabetes mellitus [31,32].

While conventional Doppler echocardiography remains a cornerstone of non-invasive cardiovascular assessment, it often proves insufficient for fully elucidating the underlying mechanisms of exertional intolerance. Consequently, cardiopulmonary exercise testing (CPET/ergospirometry) is essential to comprehensively evaluate the specific hemodynamic and ventilatory dynamics in this patient cohort.

The objective of the present study was to evaluate the echocardiographic and functional parameters in individuals living with HIV infection, specifically investigating the impact of disease duration on myocardial impairment. To achieve this, the study compared a cohort of patients with a long history of infection, (exceeding 10 years) predominantly derived from the historical Romanian pediatric cohort (infected during early childhood)—against an age-matched comparator group with a more recent diagnosis (not exceeding 8 years). This comparative framework aimed to elucidate the progressive structural changes in the myocardium to enhance diagnostic accuracy and optimize targeted therapeutic strategies.

The study protocol was reviewed and approved by the institutional Ethics Committee, and the research was conducted in strict accordance with the Declaration of Helsinki. All participants provided written informed consent prior to enrollment.

Study participants were selected exclusively from the population of PLWH, utilizing an intra-cohort comparative study design without the inclusion of a seronegative healthy control group. The primary endpoint was to assess clinical and echocardiographic disparities, based on the duration of exposure to the viral infection.

Patients were stratified into two distinct operational groups based on the time elapsed since their initial diagnosis:

- Recent-to-Intermediate Cohort: patients with a disease duration spanning between 2 and 8 years post-diagnosis.
- Long-Term Exposure Cohort: Patients with an extensive history of infection exceeding 10 years. This subpopulation integrated individuals diagnosed during adulthood as well as long-term survivors belonging to the Romanian pediatric cohort who acquired the infection during childhood [31,33].

### 4.1. Ethical Consideration

The study was conducted in strict compliance with the guidelines of Good Clinical Practice (GCP) and the ethical principles outlined in the Declaration of Helsinki regarding medical research involving human subjects [34,35]. The research protocol was prospectively evaluated and approved by the Institutional Ethics Committee of the Victor Babes Clinical Hospital of Infectious Diseases, Timisoara (approval no. 3082).

Prior to study enrollment and the execution of any clinical evaluations, echocardiographic assessments, or cardiopulmonary exercise tests, all patients were comprehensively informed regarding the research objectives, procedural protocols, and data confidentiality safeguards. Written informed consent was obtained from all participants prior to their inclusion in the study.

### 4.2. Inclusion Criteria

To ensure clinical homogeneity within the study cohort and mitigate the influence of confounding variables on myocardial parameters, stringent inclusion criteria were established. The study enrolled young and middle-aged adults, ranging from 24 to 48 years of age, who demonstrated clinical stability at the time of assessment.

A critical virological prerequisite was the efficacy of ART. Consequently, enrollment was strictly limited to patients receiving active ART with documented adherence and exhibiting sustained virological suppression. This suppression was defined as maintaining an undetectable plasma viral load (HIV-RNA < 50 copies/mL) for a minimum of 12 consecutive months prior to study inclusion.

From a cardiovascular perspective, eligibility was restricted to clinically healthy individuals with no prior diagnosis of structural or functional cardiac pathologies. The study protocol strictly excluded subjects with a history of cardiovascular events or those currently receiving chronic cardiological pharmacotherapy. Furthermore, it was mandatory for all participants to be entirely asymptomatic in their daily activities. A comprehensive anamnesis was conducted to confirm the absence of any clinical signs or symptoms indicative of heart failure or coronary artery disease, specifically confirming the absence of chest pain and pre-existing exertional dyspnea.

### 4.3. Exclusion Criteria

To ensure the accuracy of clinical evaluations and the validity of functional tests, rigorous exclusion criteria were applied, specifically tailored to the seropositive population and the prerequisites of cardiopulmonary exercise testing.

Primarily, from a virological and infectious standpoint, patients lacking viral suppression (detectable HIV-RNA viral load) and those with active opportunistic infections were excluded. Furthermore, subjects with history of severe pulmonary conditions—such as tuberculosis (TB) or *Pneumocystis jirovecii* pneumonia—were excluded, as these pathologies could induce ventilatory limitations and significantly impair respiratory exercise capacity. Similarly, individuals presenting with neurological or systemic complications associated with HIV infection were ineligible. This included HIV wasting syndrome, HIV-associated encephalopathy, posterior reversible encephalopathy syndrome (PRES), or any other neurocognitive or mental status abnormalities.

Given that pre-existing cardiovascular pathologies already constituted a non-inclusion criterion, further exclusions were aimed at eliminating major confounding factors that could influence exercise performance or the myocardial phenotype. Thus, patients with severe metabolic disorders, such as uncontrolled diabetes mellitus (according to the American Diabetes Association criteria), those with end-stage renal disease undergoing dialysis therapy, pregnant women, and patients with active oncological pathologies (including Kaposi sarcoma or HIV-associated lymphomas) were excluded. Additionally, to ensure maximum safety during testing, patients with a recent history of substance abuse or chronic alcoholism, as well as subjects with objective biomechanical limitations—such as severe musculoskeletal disorders, peripheral vascular diseases, neuromotor sequelae, and significant sensory deficits—were not enrolled.

### 4.4. Data Collection and Immuno-Virological Profiling

Demographic, clinical, and lifestyle data–including age, sex, smoking history, alcohol and recreational substance consumption, and the degree of sedentary behavior (assessed on a scale from 1 to 5 based on self-reported physical activity levels)—were systematically collected during regular outpatient monitoring visits. Crucially, the assessment of these behavioral confounders relied entirely on categorical self-report questionnaires (e.g., “current smoker vs. non-smoker”, self-rated physical activity on a scale from 1 to 5) without precise quantification of exposure intensity or duration.

To accurately characterize the immune-virological status, HIV-specific parameters were recorded: known disease duration, total duration of exposure to ART, current therapeutic regimen, nadir CD4+ T-cell count, and current absolute CD4+ T-cell count.

### 4.5. Clinical Examination and Bioimpedance Body Composition Analysis

Prior to the echocardiographic assessment, all patients underwent a comprehensive objective clinical examination, which included resting blood pressure measurement and a detailed body composition analysis. Anthropometric data and associated parameters were obtained via bioelectrical impedance analysis (BIA) using a professional, validated tetrapolar device (Tanita body composition analyzer, MC-780MA, Tanita Corp., Tokyo, Japan). To ensure quantitative accuracy and reproducibility, duplicate measurements were performed on a randomly selected subset of 15 patients, demonstrating excellent intra- and inter-operator reliability, with a coefficient of variation of <3% across all core metrics. The recorded parameters comprised: total body weight, body fat percentage, muscle mass, total body water volume, estimated bone mass, and metabolic age.

To ensure the accuracy and reproducibility of the bioimpedance measurements, the evaluation was conducted under a strict standardized protocol. Patients were instructed to adhere to the following pre-testing conditions: fasting for at least 3 to 4 h, emptying the urinary bladder immediately prior to the assessment, avoiding strenuous physical exertion for 24 h preceding the visit, and abstaining from alcohol or diuretic (caffeinated) beverages for at least 12 h before the test. The actual weighing was performed under conditions of normohydration, with patients wearing light clothing, in an upright standing position, and completely barefoot to ensure optimal contact with the analyzer’s electrodes.

Body mass index was calculated as a ratio of weight (in kilograms) to the square of height (in meters). According to the World Health Organization (WHO) criteria, patients were classified as overweight if BMI was ≥25 kg/m^2^, and obese if BMI was ≥30 kg/m^2^. Body surface area (BSA) was determined using the classic DuBois and DuBois formula; this parameter was subsequently utilized for the precise indexation of myocardial volumes and mass obtained during echocardiography [36]. Sarcopenic obesity was strictly defined combining a high BMI (≥30 kg/m^2^) with a relative reduction in appendicular skeletal muscle mass indexed to height, utilizing age- and sex-adjusted cutoffs appropriate for young adults, rather than geriatric EWGSOP2 criteria.

### 4.6. Cardiopulmonary Exercise Testing (CPET) Protocol

All cardiopulmonary exercise tests were conducted in a specialized medical center in Timisoara, Romania (Neoclinic Medical Centre, Timisoara, Romania). To eliminate interobserver variability and ensure the consistency of the acquired data, all evaluations were performed and interpreted by the same examining physician, a specialist trained and certified by the Romanian Society of Cardiology (RSC).

Functional capacity was assessed using a BLT (BLT, Ortona, Italy) motorized treadmill, applying the standardized Bruce protocol. This graded protocol was selected to allow a progressive and predictable increase in hemodynamic workload (via speed and incline adjustments every 3 min), facilitating the achievement of maximal or symptom-limited exertion. The electrical activity of the heart was monitored continuously throughout the test and the recovery period using a 12-lead electrocardiographic (ECG) system (Elite system, Micromed-Biotechnologia, Mogliano Veneto, Italy). For the safety profile and vascular response evaluation, blood pressure was measured at rest, at the end of each 3 min exercise stage, at peak exercise, and during the active and passive recovery minutes.

The analysis of respiratory gas exchange was performed in real time, on a breath-by-breath basis, throughout the entire exercise cycle utilizing the advanced Cortex (Metalyzer 3B, CPX System, Cortex Biophysik GmbH, Leipzig, Germany). To guarantee absolute accuracy of the metabolic and ventilatory measurements, equipment calibration procedures were strictly documented and executed. Gas concentration analyzers (oxygen and carbon dioxide) were calibrated using standard reference gas mixtures, and the airflow turbine was calibrated with a 3 L precision syringe immediately prior to the initiation of each CPET test.

### 4.7. Analysis of Cardiopulmonary and Echocardiographic Parameters

The analysis of ergospirometric data involved the continuous, breath-by-breath evaluation of ventilatory metabolic, and hemodynamic parameters, with mean values calculated at 30 s intervals. Peak oxygen uptake (peak VO_2_) was defined as the highest value recorded over a 20 s period of maximal exertion. To validate the achievement of maximal effort, the respiratory exchange ratio was continuously monitored. The assessment of the hemodynamic response to exercise included the calculation of the oxygen pulse (VO_2_/HR) —serving as an indirect surrogate for stroke volume—alongside the analysis of the heart rate reserve and peak circulatory power (defined as the product of peak VO_2_ and maximal systolic blood pressure).

In contrast to conventional visual estimators, the anaerobic threshold (AT) was objectively determined using the V-Slope method, which analyzes the linear relationship between carbon dioxide production (VCO_2_) and oxygen consumption (VO_2_). Ventilatory dynamics and metabolic efficiency were rigorously evaluated by calculating specific linear regression slopes, including: the VCO_2_/VO_2_ slope, the oxygen uptake efficiency slope (OUES), the dynamics of oxygen consumption relative to workload (the ΔVO_2_/ΔWatt ratio), and the heart rate response curve (the ΔHR/ΔVO_2_ ratio). Ventilatory efficiency was further quantified using the ventilatory equivalents for oxygen and carbon dioxide (VE/VO_2_ and VE/VCO_2_, respectively).

Concurrently, to correlate exercise performance with resting cardiac function, a detailed echocardiographic analysis was performed. This included the determination of the left ventricular ejection fraction (LVEF) via the biplane Simpson’s method, as well as an advanced assessment of myocardial mechanics through the measurement of global longitudinal strain (GLS). Diastolic function was quantified by evaluating the transmitral flow velocity (the E/A ratio) and myocardial tissue velocities via tissue Doppler imaging (TDI) to derive the E/e’ ratio, alongside the determination of indexed left atrial volumes and the estimation of pulmonary artery systolic pressure (PASP).

### 4.8. Exercise Test Termination Criteria

For reasons of strict clinical safety, the protocol mandated the immediate termination of testing upon the occurrence of any of the following criteria: a severe hypertensive response (systolic blood pressure > 260 mmHg and/or diastolic blood pressure > 140 mmHg); a hypotensive response (a decrease in systolic blood pressure of >20 mmHg below resting levels; electrocardiographic changes suggestive of myocardial ischemia (ST-segment depression > 2.0 mm, T-wave inversion, or de novo Q-wave development); malignant arrhythmias (sustained supraventricular or ventricular tachycardia); the onset of anginal chest pain; symptoms of cerebral hypoperfusion (presyncope, syncope, disorientation, or loss of coordination); severe dyspnea disproportionate to the exercise workload; or marked pallor or excessive diaphoresis.

### 4.9. Transthoracic Echocardiography and Tissue Doppler Imaging (TDI)

#### 4.9.1. Examination Conditions and Image Acquisition

Echocardiographic evaluations were performed at a specialized cardiovascular center (Neoclinic Medical Center, Timisoara, Romania) by the same senior cardiologist, who possesses advanced expertise in echocardiography. All examinations were conducted under resting conditions using standardized imaging protocols in strict adherence to the current guidelines set forth by the American Society of Echocardiography (ASE) and European Association of Cardiovascular Imaging (EACVI). Furthermore, to validate quantitative accuracy and eliminate observer bias, a randomly selected subset of echocardiograms (20%) was independently re-evaluated by a second blinded imaging specialist. Inter-reader concordance was excellent, yielding an interclass correlation coefficient (ICC) of >0.90 for key parameters, including left ventricular mass index and Global Longitudinal Strain. M-mode, two dimensional (2D), and Doppler (pulse-wave, continuous-wave, color), and tissue Doppler imaging data were acquired from standard parasternal and apical acoustic windows.

#### 4.9.2. Structural, Volumetric, and Systolic Function Analysis

Chamber dimensions and wall thicknesses were evaluated using M-mode and 2D echocardiography (Teichholz method) to determine interventricular septal (IVS) thickness, left ventricular posterior wall (LVPW) thickness, and left ventricular internal diameter (LVID). For the assessment of LV volumes and global systolic function, the biplane Simpson’s volumetric method was utilized from the apical four-chamber (A4C) view, allowing the determination of the following parameters:

- Left ventricular end-diastolic volume (LVEDV) and end-systolic volume (LVESV);
- Stroke volume (SV) and left ventricular ejection fraction (LVEF);
- Left atrial (LA) volume and LA area.

Additionally, for a refined assessment of subclinical myocardial dysfunction, myocardial deformation was evaluated by calculating the global longitudinal strain (GLS).

#### 4.9.3. Diastolic Function and Hemodynamic Assessment

LV diastolic function was comprehensively analyzed by combining transmitral inflow parameters (via pulsed-wave Doppler) with TDI measurements at the level of the mitral annulus. The recorded parameters included:

- From transmitral flow: Peak early diastolic velocity (E-wave), peak late diastolic velocity (A-wave), the E/A ratio, and deceleration time (DT)
- From tissue Doppler imaging: Early diastolic (e′) and late diastolic (a′) mitral annular velocities, alongside the calculation of the E/e′ ratio, a pivotal surrogate parameter for estimating LV filling pressures.

Furthermore, PASP was estimated by interrogating the tricuspid regurgitation jet velocity via Doppler and measuring the peak pressure gradient (peak PG).

#### 4.9.4. Complementary Vascular Evaluation

To complement the comprehensive cardiovascular risk profile, the ultrasonographic protocol also included the evaluation of the internal carotid arteries (ICA, right and left) to detect the presence of atheromatous plaques and assess their subsequent hemodynamic significance.

### 4.10. Cardiopulmonary Exercise Testing (CPET/Ergospirometry)

#### 4.10.1. Functional Capacity and Cardiorespiratory Assessment

The evaluation of functional capacity and the cardiorespiratory response to exercise was performed via cardiopulmonary exercise testing utilizing a motorized treadmill ergometer. The testing procedures were directly supervised by the same specialized physician, ensuring continuous monitoring of the ECG tracings, heart rate (HR), and blood pressure (both systolic and diastolic). Respiratory gas exchange was analyzed continuously on a breath-by-breath basis, facilitating the extraction of the following core parameters regarding aerobic performance and ventilatory efficiency:

- Peak oxygen uptake (peak VO_2_): Expressed both as an absolute value (mL/min) and relative to total body weight (mL/kg/min), and subsequently reported against age- and sex-predicted normative values.
- Carbon dioxide production and Respiratory Exchange Ratio: Continuously monitored and utilized to validate the achievement of maximal physical exertion. Verification of maximal effort was stringently defined by objective criteria, specifically the attainment of a respiratory exchange ratio ≥ 1.10 and the achievement of ≥85% of the age-predicted heart rate reserve (HRR).
- Oxygen pulse: Serving as an indirect surrogate marker for stroke volume dynamics during exertion.
- Minute ventilation (VE) and breathing frequency (BF).

#### 4.10.2. Determination of Ventilatory Thresholds and Slopes

The anaerobic threshold (AT) was determined independently and objectively by employing the V-Slope method. For a comprehensive evaluation of ventilatory efficiency and metabolic response dynamics, specific linear regression slopes were calculated, which included:

- The minute ventilation to carbon dioxide production slope (VE/VCO_2_ slope)
- The oxygen uptake efficiency slope (OUES)
- The dynamics of oxygen consumption relative to the generated work rate (the ΔVO_2_/Δ Watt ratio).

### 4.11. Statistical Analysis

Statistical analysis was performed using IBM SPSS Statistics software, version 26.0 (IBM Corp., Armonk, NY, USA). The primary database was compiled and structured in Microsoft Excel (Microsoft Corporation, Redmond, WA, USA). The distribution of continuous variables was assessed for normality using the Shapiro–Wilk test: normally distributed continuous variables were expressed as mean ± standard deviation; non-normally distributed continuous data were presented as median and interquartile range (IQR) and categorical variables were reported as absolute frequencies and percentages.

Comparative Analysis: to compare the two operational study groups (patients with recent infection versus those with a long-standing history/pediatric cohort), the following statistical tests were applied:

- The unpaired Student’s t-test was utilized for normally distributed continuous variables. In instances where the assumption of homogeneity of variances was violated (as evaluated by Levene’s test), Welch’s correction was applied.
- To maintain statistical rigor for non-parametric continuous data, the Mann–Whitney U test was employed.
- Associations between categorical variables were analyzed using the Chi-square (X^2^) test or Fisher’s exact test, as appropriate, to ensure the mathematical robustness of the comparisons.
- To adjust for confounding factors in group comparisons (such as the impact of disease duration on metabolic variables across different ART classes), ANCOVA models were utilized.

Correlation and Regression Analyses: To evaluate correlations between functional echocardiographic parameters (e.g., the E/e’ ratio, GLS) and cardiopulmonary exercise testing variables, the Pearson correlation coefficient (for normally distributed data) or Spearman’s rank correlation coefficient (for non-parametric data) was utilized.

Furthermore, to identify independent predictors of subclinical myocardial impairment or reduced functional exercise capacity, and to control for potential confounding factors (age, BMI, smoking status, ART duration, and nadir CD4+ count), a multivariate logistic regression analysis was conducted. The results were reported as adjusted odds ratio (aOR) with 95% confidence intervals (CI).

Data management followed a strict complete-case analysis protocol; to ensure statistical integrity, only participants with comprehensive sets of clinical and investigational data were included in the final analysis. A two-sided *p*-value < 0.05 was considered statistically significant.

## 5. Conclusions

The findings of this study provide a comprehensive characterization of the cardiometabolic phenotype in a cohort of 50 virologically suppressed PLWH, highlighting a profound dissociation between successful ART-mediated viral suppression and persistent subclinical cardiovascular and metabolic impairment. Our results illustrate a fundamental paradigm shift in the clinical management of HIV: while the pre-ART era was dominated by opportunistic myocardial infections and severe dilated cardiomyopathies, the contemporary era is defined by a “functional and metabolic footprint” characterized by accelerated biological aging and structural metabolic dysregulations.

Several critical insights emerge from our data:

- Sarcopenic Obesity as a Functional Determinant: We identified sarcopenic obesity—the synergy of reduced skeletal muscle mass and increased visceral adiposity—as a more robust predictor of exercise intolerance than standard lipid parameters alone. This structural remodeling suggests that modern cardiovascular risk in PLWH is increasingly driven by metabolic phenotypic changes rather than purely iatrogenic pharmacological toxicity.
- The Impact of Contemporary Regimens: The prevalence of INSTI-based regimens, while effective for virological control, correlates significantly with increased visceral fat ratings and BMI. This suggests that modern therapeutic strategies may inadvertently exacerbate the metabolic burden, necessitating a more granular approach to metabolic monitoring.
- “Functional HIV-Associated Frailty”: Our integration of cardiopulmonary exercise testing revealed that the “Metabolic Age Gap” and the TG/HDL-cholesterol ratio are high-utility clinical surrogates for insulin resistance and cardiovascular risk. These markers identify a subset of patients who exhibit “functional HIV-associated frailty”—a state of cardiopulmonary incompetence that manifests as premature anaerobic threshold attainment, years before the clinical onset of HFpEF.
- The Persistence of Historical Imprinting: The strong correlation between nadir CD4+ counts and accelerated biological aging indicates that historical immunodeficiency leaves a permanent functional imprint. Current immune reconstitution, however robust, does not fully mitigate this cumulative pathological burden.

In conclusion, while our findings strongly advocate for a paradigm shift from a purely viral-centric model to an integrated, cardiometabolic approach, we must emphasize that this proposed monitoring framework is currently based on cross-sectional associations. The routine implementation of CPET, along with body composition analysis and metabolic surrogate markers like the TG/HDL ratio, is essential for identifying high-risk individuals. Early detection of subclinical functional impairment is paramount to innovating targeted therapeutic interventions and optimizing long-term cardiovascular outcomes in an aging HIV-positive population. However, without evidence of temporal precedence, clinical translation of this approach requires prospective validation in cohorts with repeated phenotyping and pre-specified cardiovascular endpoints to confirm whether mitigating metabolic frailty actively prevents overt HFpEF. Future prospective studies are required to validate these metabolic markers as prognostic tools for cardiovascular morbidity in the era of modern antiretroviral therapy.

## Figures and Tables

**Table 1:** Comparative Analysis of Anthropometric, Metabolic, and Functional Parameters Stratified by HIV Exposure Duration.

Parameter	Recent-to-Intermediate (<10 Years) (*n* = 25)	Long-Term Exposure (≥10 Years) (*n* = 25)	*p*-Value
*BMI* (kg/m^2^)	23.8 ± 3.2	26.6 ± 4.4	0.032
*LDL Cholesterol* (mg/dL)	118 ± 28	152 ± 52	0.015
*VO*_2_ *peak (% predicted)*	78.4 ± 12.5	46.4 ± 10.2	<0.001
*VE/VCO*_2_ *Slope*	28.5 ± 3.2	36.3 ± 4.5	0.004
*Metabolic Age* vs. *Chronological Age*	1.8 ± 1.2	7.2 ± 3.5	0.002

**Table 2:** Multivariate Regression Analysis: Independent Predictors of Reduced Aerobic Capacity (VO_2_ peak < 65% of predicted value).

Predictor Variable	Adjusted Odds Ratio (aOR)	95% CI	*p*-Value
*Global Longitudinal Strain (GLS)*	0.72	0.58–0.89	0.003
*Triglyceride/HDL Ratio*	2.14	1.15–3.98	0.016
*Nadir CD4+ Count*	0.998	0.991–0.999	0.021
*Visceral Fat Rating*	1.45	1.08–1.95	0.012
*Sarcopenic Obesity (Binary)*	2.85	1.22–6.65	0.010

## Data Availability

The authors confirm that all data points, including immunological ratios and bioimpedance metrics, are derived from actual patient measurements. The raw, identified source data supporting the conclusions of this manuscript can be provided upon request to the corresponding author (due to internal regulations of the hospital—Regulation UE nr. 679 from 2016 regarding protection of personal data).

## Abbreviations

The following abbreviations are used in this manuscript:

ART	Antiretroviral Therapy
ASE	American Society of Echocardiography
A4C	Apical Four-Chamber
ANCOVA	Analysis of Covariance
ANOVA	Analysis Of Variance
aOR	Adjusted Odds Ratio
AT	Anaerobic Threshold
BF	Breathing Frequency
BIA	Bioelectrical Impedance Analysis
BMI	Body Mass Index
BSA	Body Surface Area
CI	Confidence Interval
CPET	Cardiopulmonary Exercise Testing
CVDs	Cardiovascular Diseases
EACVI	European Association of Cardiovascular Imaging
ECG	Electrocardiographic
EPV	events-per-variable
EWGSOP2	European Working Group on Sarcopenia in Older People 2
GCP	Good Clinical Practice
GLS	Global Longitudinal Strain
HDL	High Density Lipoprotein
HF	Heart Failure
HFpEF	Heart Failure with Preserved Ejection Fraction
HIV	Human Immunodeficiency Virus
HR	Heart Rate
HRR	Heart Rates Reserve
INSTI	Integrase Strand Transfer Inhibitor
ICC	interclass correlation coefficient
IQR	Interquartile Range
IR	Insulin Resistance
IVS	Interventricular Septal
LA	Left Atrial
LDL	Low-Density Lipoprotein
LV	Left Ventricular
LVEF	Left Ventricular Ejection Fraction
LVEDV	Left Ventricular End-Diastolic Volume
LVESV	Left Ventricular End-Systolic Volume
LVID	Left Ventricular Internal Diameter
LVPW	Left Ventricular Posterior Wall
NNRTI	Non-Nucleoside Reverse Transcriptase Inhibitor
OR	Odds Ratio
OUES	Oxygen Uptake Efficiency Slope
PASP	Pulmonary Artery Systolic Pressure
PI	Protease Inhibitor
PLWH	People Living With HIV
PRES	Posterior Reversible Encephalopathy Syndrome
RER	Respiratory Exchange Ratio
RSC	Romanian Society of Cardiology
SD	Standard Deviation
STR	Single-Tablet Regimens
SV	Stroke Volume
TB	Tuberculosis
TDI	Tissue Doppler Imaging
TG	Triglycerides
TG/HDL	Triglycerides/High Density Lipoprotein
UNAIDS	Joint United Nations Program On HIV
VE	Minute Ventilation
VO_2_ peak	Peak Oxygen Uptake
WHO	World Health Organization
